# Effect of Incorporation of Zeolite Containing Silver-Zinc Nanoparticles into Mineral Trioxide Aggregate on Odontogenic Activity of Human Dental Pulp Stem Cells

**DOI:** 10.30476/DENTJODS.2020.86183.1172

**Published:** 2021-09

**Authors:** Negin Ghasemi, Sadegh Salarinasab, Reza Rahbarghazi, Samin Sedghi, Paria Davoudi

**Affiliations:** 1 Dept. of Endodontics, Dental and Periodontal Research Center, Dental Faculty, Tabriz University of Medical Sciences, Tabriz, Iran; 2 Institute of Basic and Clinical Physiology Sciences, Kerman University of Medical Science, Kerman, Iran; 3 Dept. of Applied Cell Sciences, Faculty of Advanced Medical Sciences, Tabriz University of Medical Sciences, Stem Cell Research Center, Tabriz University of Medical Sciences, Tabriz, Iran; 4 Private Office, Tabriz, Iran; 5 Dept. of Endodontics, Dental Faculty, Shahid Beheshti Medical University, Tehran, Iran

**Keywords:** Alkaline phosphatase, Biomineralization, Dental pulp stem cells, Nanoparticles, Zeolite

## Abstract

**Statement of the Problem:**

The stimulation of odontogenic activity is considered an essential property for biomaterials used in vital pulp therapy.

**Purpose:**

The present study aimed to evaluate the effect of the incorporation of zeolite containing silver-zinc nanoparticles (Ze-Ag-Zn) into Angelous mineral trioxide aggregate (AMTA) on the odontogenic activity of human dental pulp stem cells (HDPSCs).

**Materials and Method:**

In this *in vitro* study, HDPSCs were treated with 2% wt of synthesized Ze-Ag-Zn particles+AMTA, AMTA and Ze-Ag-Zn disks. The negative control cells did not receive any treatment. Then, cell viability was measured using the MTT assay after 7 and 14 days of the treatment course. The alkaline phosphatase (ALP) activity and calcium ion level were also measured in the supernatant culture media using auto-analyzer kits. The obtained data were analyzed using one-way ANOVA and Student t-test where appropriate. The level of the statistical significance was set at *p*< 0.05.

**Results:**

The results indicated that HDPSCs treated with AMTA and AMTA+Ze-Ag-Zn particles did not show any significant cell death compared with the control cells after 14 days of the treatment course while the ALP activity and calcium ion levels were significantly (*p*< 0.05) elevated. Also, the addition of AMTA particles to the cell culture media resulted in increased ALP activity and calcium ion level compared with HDPSCs treated with AMTA+ Ze-Ag-Zn particles on day 7 of the treatment course (*p*< 0.05).

**Conclusion:**

It seems that the incorporation of Ze-Ag-Zn particles into AMTA did not have any significant positive effect on the biomineralization properties of AMTA.

## Introduction

The ability to induce biomineralization along with their biocompatibility is regarded as featured characteristics of the materials used for maintaining or regenerating a vital pulp [ 1 ]. Mineral trioxide aggregate (MTA) and other types of calcium-silicate based cement are broadly utilized in this field due to their biocompatibility and potential for the development of mineralized tissues [ 2 - 3 ]. However, there are few drawbacks to some of these products like MTA such as extended setting time and poor management [ 2 ]. During the last years, many efforts have been made to improve the properties of MTA [ 4 - 5 ], like incorporation of zeolite containing silver-zinc nanoparticles (Ze-Ag-Zn).

Zeolite is the crystalline and microporous structure of alumino-silicate, which is capable of carrying some ions, such as silver or zinc. The reason for its application originates from its porous structure, high absorption capacity, and the ion exchange properties [ 6 ] that led to beneficial effects such as antimicrobial potential [ 7 - 8 ]. In addition, incorporation of 2% wt of zinc and silver particles into MTA resulted in improved antibacterial activity [ 9 ], increased solubility, and decreased setting time [ 10 ], with no considerable cytotoxic effects [ 11 - 12 ]. However, two recent studies have revealed that this combination decreased the compressive and push-out bond strength of MTA [ 11 , 13 ]. 

It is noteworthy that zinc, as an essential trace element, participates in intracellular signaling pathways and has stimulatory effects on osteo/odontogenesis [ 8 ]. It has been reported that eluted forms of zinc ions increase the expression of osteoblast markers, including alkaline phosphatase (ALP), type I collagen, osteocalcin and calcium deposition in dental pulp stem cells [ 14 ]. Moreover, it has been shown that silver nanoparticles can promote mesenchymal stem cells and osteogenic differentiation via the induction of TGF-β/BMP signaling in these types of cells [ 15 ].

There are no data about the biomineralization capacity of Ze-Ag-Zn particles when incorporated into MTA; thus, in the current in vitro study we investigated the impact of incorporating 2% wt Ze-Ag-Zn particles into MTA on the odontogenic activity of human dental pulp stem cells (HDPSCs) via assessing the ALP activity and calcium ion levels.

## Materials and Method

### Synthesis of Ze-Ag-Zn Particles

HZSM-5 zeolite was manufactured in the laboratory. Zinc nitrate, Zn(NO3)2, and silver nitrate (AgNO_3_), was purchased from Merck (Merck, Darmstadt, Germany). The powder form of ZSM-5 was altered by liquid phase ion exchange (LPIE), using Ag+ and Zn2+ cations. Following the preparation of Ag (I)-ZSM-5, by 24-h ion exchange with a solution of 10g of ZSM-5 and 300mL of AgNO_3_ (1M) at ambient temperature, the preparation of Ag (I) and Zn (II)-ZSM-5(Ze-Ag-Zn nanoparticles) was performed by adding 10g of Ag (I)-ZSM-5 to 300 mL of Zn(NO3)2 solution (5M). After each exchange process, the modified zeolite suspension was filtered and washed with profuse amounts of deionized water [ 16 ].

### Characterization of Synthesized Ze-Ag-Zn Particles

Scanning electron microscopy (Model: MRIA3-FEG-SEM Tescan, Brno, Czech) and transmission electron microscopy (Zeiss LEO 912 Omega) were used to classify the morphological features of the synthesized Ze-Ag-Zn particles. 

**Figure 1:**
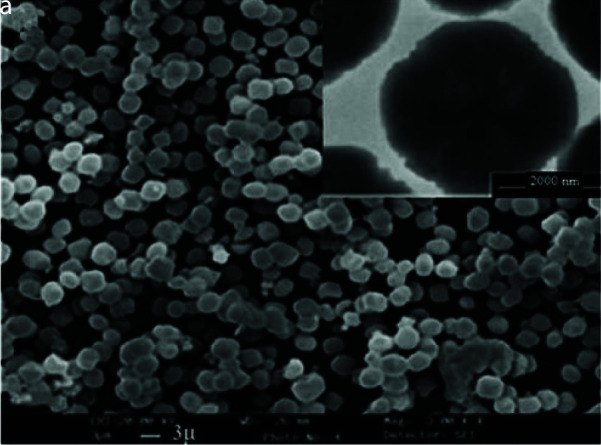
The size and morphology of Ze-Ag-Zn particles. The average of the particle diameter of the composite is about 2-3 μm

As depicted in Figure 1, Ze-Ag-Zn crustal composites exhibit spherical to cubical shapes with no noticeable amorphous phase, denoting high purity of samples. 

### Sample Preparation

In this study, HDPSCs were treated with Angelus MTA (Angelus, Londrina, Brazil) (AMTA), AMTA + Ze-Ag-Zn particles (AMTA+ Ze-Ag-Zn) in which 2%wt of Ze-Ag-Zn particles was added to AMTA, and the negative control group (plastic surface) in which the cells receive no treatment. The materials were combined according to the instructions of the manufacturer and packed as disks of varying diameters on 96- and 24-well plates and 2 mm thick under aseptic conditions, covered in wet gauze bits and incubated for 24 hours in a closed container. Following the sterilization of the disks with ethylene dioxide gas, they were placed on the bottom surface of each well of the plates, and the cells were cultured to examine the effect of various treatments after washing twice with phosphate-buffered saline.

### Cell Culture

HDPSC-line was procured from the Royan Institute, Tehran, Iran. HDPSCs were placed in low-content glucose Dulbecco’s modified Eagle medium (Gibco), supplemented with 10% fetal bovine serum (Gibco) and 1% penicillin-streptomycin (Biosera). The culture plates were incubated at 37°C under a humidified atmosphere with 5% CO_2_. Various treatments were conducted on cells extracted from the third to sixth passage.

### MTT Assay

The cytotoxic impact of various treatments on HDPSC's viability was calculated with the colorimetric test (4,5-dimethylthiazol-2–yl),2.5-diphenyltetrazo-lium bromide. Mosmann’s tetrazolium toxicity (MTT) assay (Sigma Chemical Co., St Louis, MO, USA) was employed in this study. For this purpose, 1 mL of the low-content glucose Dulbecco’s modified Eagle medium along with 1% fetal bovine serum containing 1×10^5^ HDPSCs was transferred to each well of the 96-plate coated with disks at the bottom of the wells. The negative control cells were poured into blank wells. The supernatants of the cell culture media were discarded on days 7 and 14 and the disks were incubated for 4 hours with 100μL of MTT (5mg/mL). Subsequently, 50μL of dimethyl sulfoxide was applied to each well and the optical absorbance of the samples was read at a wavelength of 450 nm using a microplate reader (BioTek). The viability percentage in relation to control cells was calculated as the cell percentage. 

### Alkaline Phosphatase Activity

The activity of ALP in HDPSCs treated with various treatments was determined using an auto-analyzer kit (Roche Hitachi 912, Germany). In this method, 1×10^5^ HDPSCs were seeded onto the 24-well plates containing 2 ml of the culture medium. The supernatant was extracted and centrifuged at 400 g for 5 minutes on days 7 and 14 of the treatment course. First, the activity of the ALP was measured and represented as IU/ L. 

### Calcium Ion Level

For the assessment of different treatments on the odontogenic activity of HDPSCs, the level of calcium ion was analyzed in the supernatant of the cell culture media. On days 7 and 14, following the seeding of 1×10^5^ HDPSCs onto the 24-well plates, the supernatant was collected and centrifuged at 400 g for 5 minutes. Finally, the total calcium ion content was evaluated using an auto-analyzer (Roche Hitachi 912, Germany) and expressed as mg/L.

### Statistical Analysis

All in vitro experiments were performed triplicates. The obtained values were expressed as the means and standard deviation (mean±SD). The analysis of the data was conducted by SPSS software version 20.0 (SPSS Inc., Chicago, IL). The difference between the various treatment protocols was analyzed by one-way analysis of variance (ANOVA) followed by Tukey’s post hoc test. The level of the statistical significance was set at *p*< 0.05. 

## Results

### Cell Viability

**Figure 2:**
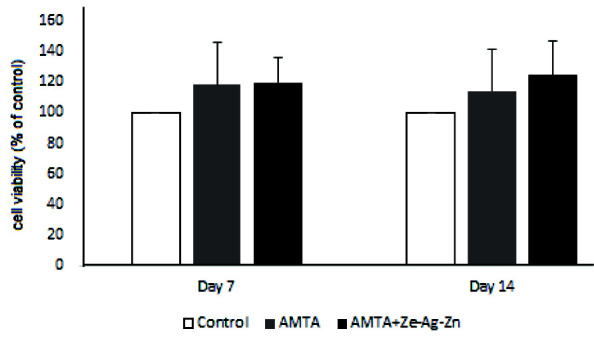
Cell viability of HDPSCs (Human Dental Pulp Stem Cells) after exposure to test materials over a period of 14 days

Based on the data extracted from the MTT test, the cell viability rate in HDPSCs treated with AMTA and AMTA+ Ze-Ag-Zn increased over a 14-day period relative to the control group but this increase was not statistically significant over a 14-day period (Figure 2).

### Alkaline Phosphatase Activity

**Figure 3:**
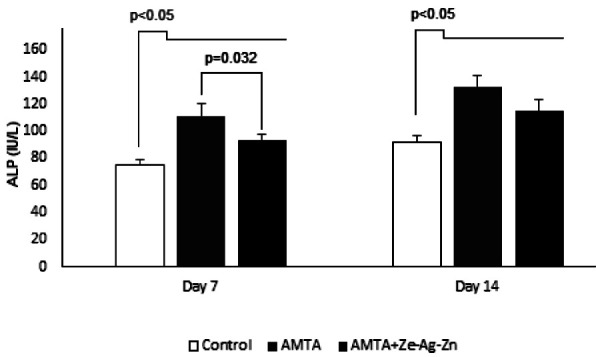
ALP (Alkaline Phosphatase) activity in experiment groups

The statistical analysis revealed that the increase in ALP activity of HDPSCs treated with AMTA and AMTA+ Ze-Ag-Zn were statistically significant (*p*<0.05) relative to the control group on days 7 and 14. In addition, the activity of ALP was significantly higher in HDPSCs treated with AMTA on day 7 than that of those treated with AMTA + Ze-Ag-Zn (*p*< 0.05; Figure 3).

### Ionized Calcium Level

**Figure 4:**
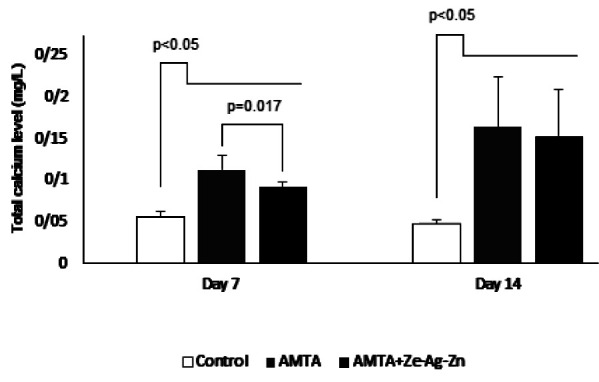
Calcium ion level in experiment groups

Related to changes in ALP activity, there was a statistically significant (*p*< 0.05) rise in calcium ion concentrations in HDPSCs treated with AMTA and AMTA+Ze-Ag-Zn on days 7 and 14.The concentrations of the calcium ion were significantly higher in HDPSCs treated with AMTA on day 7 than those treated with AMTA+ Ze-Ag-Zn (*p*< 0.05; Figure 4).

## Discussion

Currently, clinicians broadly use MTA as an endodontic biomaterial in various scientific fields such as vital pulp therapy or regeneration, regarding its ability in osteo/odontogenesis and biocompatibility [ 2 ]. However, some drawbacks of this material have led to the development of new formulations through the addition of some particles, such as zirconium oxide, zinc oxide, and silver zeolite, to improve its features. In this *in vitro* research, we evaluated the impact of silver-zinc zeolite nanoparticles in combination with AMTA on the odontogenic activity of HDPSCs.

In the present study, 2 wt% Ze/Ag/Zn composites were incorporated into MTA powder. Such percentage of silver zeolite particles has the optimum properties in terms of solubility, the release of calcium ions, antimicrobial activity, and setting time for MTA according to previous results [ 9 - 10 ].

Since the materials used in this study were in direct and permanent contact with the pulp or periapical tissues, the low cytotoxicity of the materials was predictable. In the present study, similar to HDPSCs treated with AMTA alone, cells treated with Ze-Ag-Zn particles incorporated into AMTA showed no significant difference in the cell survival rate in comparison with the control cells. Consistent with our findings, a recent study conducted by Samiei *et al*. [ 11 ] demonstrated that Ze-Ag-Zn particles have no considerable toxicity on the cells up to 5mg/mL. Moreover, other studies have reported the biocompatibility of silver and zinc nanoparticles in particular concentrations when combined with MTA and Portland cement, respectively [ 17 - 18 ]. However, excessive concentrations of Zn promote reactive oxygen species (ROS) production and induce membrane dysfunction [ 19 - 20 ]. 

MTA and other types of calcium silicate-based cement play a significant role in regeneration and remineralization, as well as the promotion of hydroxyapatite formation, which is started by calcium ion release from these materials [ 2 ]. Moreover, ALP is a specific functional enzyme expressed during osteogenic/ odontogenic differentiation and plays an essential role in tissue mineralization [ 21 ]. In this study, we found that the incorporation of 2% wt Ze-Ag-Zn particles into AMTA was not able to increase ALP activity as well as the release of calcium ions in comparison with AMTA; however, the increase in ALP activity and calcium ion release was significantly higher than the control cells. Moreover, the increase in the rate of ALP activity and calcium ion level in HDPSCs with AMTA was more pronounced than those treated with AMTA+Ze-Ag-Zn on day 7. These results seem to be in contrast with the increased solubility of the materials in response to the addition of silver zeolite particles, as reported by Çinar *et al*. [ 10 ]. However, it should be noted that, in agreement with our results, they failed to show a significant difference in the level of calcium ions. On the other side, studies have demonstrated the increased levels of osteogenic factors, ALP activity, and the deposition of calcium mineral in response to the treatment of cells with zinc oxide nanoparticles [ 14 , 22 - 23 ]. It has been shown that zeolite possesses osteogenic capacity when exposed to direct contact with cells [ 6 ]. The presence of some limitations in our study caused some different results, such as the form of materials used, which might be better to use the extract form of the materials for higher efficiency of interactions with the cells.

## Conclusion

It seems that incorporating Ze-Ag-Zn particles into AMTA did not have a significant role in the biomineralization ability of this material. Regarding the results of existing studies and the limitations in this regard, further studies are needed to evaluate the underlying interaction between HDPSCs and these types of particles at a molecular level.
